# How to assess the value of low-value care

**DOI:** 10.1186/s12913-020-05825-y

**Published:** 2020-11-02

**Authors:** José Antonio Sacristán

**Affiliations:** Department of Preventive Medicine and Public Health, School of Medicine, Universidad Autónoma de Madrid, Avenida Arzobispo Morcillo s/n, 28029 Madrid, Spain

**Keywords:** Value, Cost-effectiveness, Efficiency, Comparative effectiveness

## Abstract

**Background:**

Many of the strategies designed to reduce “low-value care” have been implemented without a consensus on the definition of the term “value”. Most “low value care” lists are based on the comparative effectiveness of the interventions.

**Main text:**

Defining the value of an intervention based on its effectiveness may generate an inefficient use of resources, as a very effective intervention is not necessarily an efficient intervention, and a low effective intervention is not always an inefficient intervention. The cost-effectiveness plane may help to differentiate between high and low value care interventions. Reducing low value care should include three complementary strategies: eliminating ineffective interventions that entail a cost; eliminating interventions whose cost is higher and whose effectiveness is lower than that of other options (quadrant IV); and eliminating interventions whose incremental or decremental cost-effectiveness is unacceptable in quadrants I and III, respectively. Defining low-value care according to the efficiency of the interventions, ideally at the level of subgroups and individuals, will contribute to develop true value-based health care systems.

**Conclusion:**

Cost-effectiveness rather than effectiveness should be the main criterion to assess the value of health care services and interventions. Payment-for-value strategies should be based on the definition of high and low value provided by the cost-effectiveness plane.

## Background

Most Western health systems are trying to identify and reduce wasteful health care use. Apart from avoiding potentially harmful treatments, the estimated cost of waste in countries like the US ranges from $760 billion to $935 billion, accounting for approximately 25% of total health care spending [1]. Surprisingly, many of the strategies designed to reduce low-value care have been implemented without a consensus on the definition of the term “value”. For example, the list of low-value services published by the Choosing Wisely campaign, an initiative led by the American Board of Internal Medicine (ABIM) Foundation, which coordinated more than 50 medical specialty societies, includes “services that provide little or no health benefit to patients” [2]. This means that the list has been drawn up applying the criteria of effectiveness and not that of efficiency. For example, out of the 435 health services included in Choosing Wisely, only 2% cite cost-effectiveness to justify the recommendations, and only 29% of criteria for services contain the word “cost” (or related terms) vs 68% of criteria for services containing the words “clinical”, “outcome”, or “harm” [3]. In the same way, the American Society of Clinical Oncology (ASCO) has recently established effectiveness (without considering costs) as a key prioritization criterion. For example, a survival gain lower than 2.5 to 3 months would not be considered a clinically meaningful outcome for squamous cell lung cancer [4].

## Main text

Prioritizing based on effectiveness might seem like a good saving strategy. However, it is well known that value and savings are not two equivalent concepts, and neither are “effectiveness” and “efficiency”. According to the well-known definition of value coined by Michael Porter, “*achieving high value for patients must become the overarching goal of health care delivery, with value defined as the health outcomes achieved per dollar spent*” [5]. This definition of value in terms of outcomes relative to costs, is a direct reference to the classic concept of efficiency.

According to the previous definition, it does not make sense to select a low-value intervention considering only its effectiveness and not its cost. Continuing with the previous ASCO example, why the health care system should reject an intervention that is only slightly more effective than another one without considering the cost of the two options? The challenge to achieve value-based prioritization is not to add the cost-effectiveness criterion to the list of low-value interventions, as suggested by some authors [3], but to replace the effectiveness criterion by the efficiency criterion. Including effectiveness in value estimates can generate inefficient use of resources, since a very effective intervention is not necessarily an efficient intervention, and a low effective intervention is not always an inefficient intervention [6].

Any decision on the value (or efficiency) of an intervention must be linked to the concept of opportunity cost. The choice of a health service necessarily detracts resources that could be used to finance other options. An intervention may be considered efficient if the resources invested to achieve a certain additional benefit cannot be used in another option that generates a greater benefit. For this reason, some of the recent proposals designed to assess the value of specific health care interventions, such as Multiple Criteria Decision Analysis (MCDA) [7], or Value Frameworks developed by different organizations for determining the value of cancer drugs [8], are not appropriate tools as they ignore opportunity cost [9].

Despite its well-known limitations [10], cost-effectiveness analysis, based on cost of opportunity, remains the cornerstone for analyzing the value of a health intervention [11]. Although it is not the objective of this work to carry out a comprehensive review of its theoretical bases, it is worth it to remember that the economic evaluation of health interventions is a method proposed more than 40 years ago by Weinstein and Zeckhauser [12], being their most important contribution the creation of a unit to measure health outcomes, the Quality Adjusted Life Year (QALY), which combined survival and quality of life (or utility). Apart from standardizing the health outcomes measures of different types of interventions, the QALY can be used to calculate the incremental cost-effectiveness ratio (ICER), that is the additional cost per additional unit (QALY) of benefit gained [13].

**Fig. 1:**
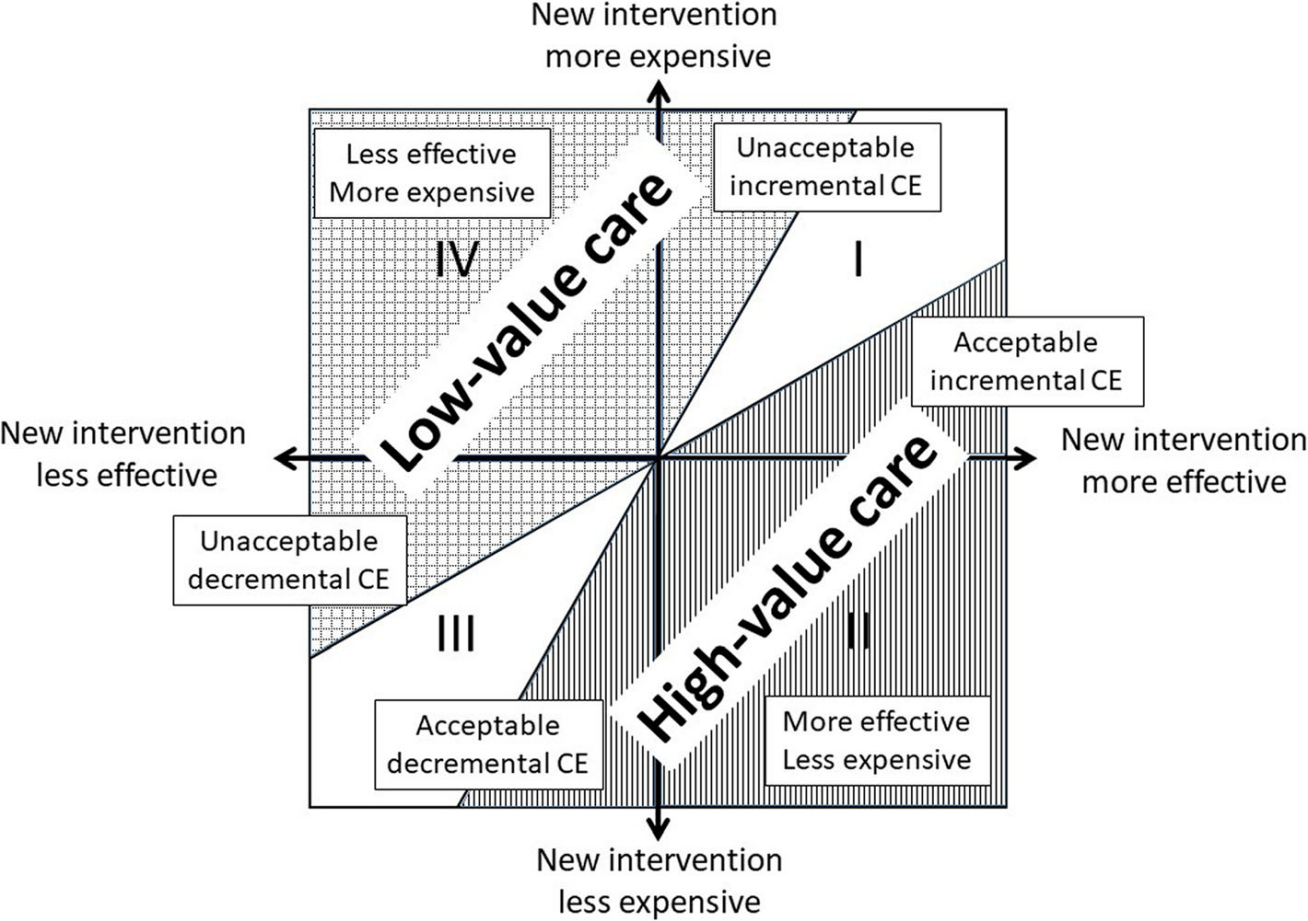
High and low value care areas in the cost-effectiveness plane

The relationship between cost and effectiveness of alternative interventions can be represented as a cost-effectiveness plane (Fig. 1). Interventions that are more expensive but less effective (quadrant IV) should be rejected, while interventions that are more effective but less expensive (quadrant II) should be adopted. The decisions are not so obvious in quadrants I and III. In quadrant I, where interventions are more effective and more expensive, it is necessary to define efficiency thresholds that indicate how much the health system is willing to pay in relation to the anticipated increase in benefit. Organizations such as NICE in the United Kingdom or ICER in the US have defined a double threshold system that facilitates the gradualness of decisions. For example, the 20,000 and 30,000 pound-thresholds per QALY defined by NICE indicate that, below 20,000 pounds per QALY, the intervention is likely to be adopted, while above 30,000 pounds per QALY, it is normal to be rejected [14]. The lower limit proposed by ICER for the US is $ 50,000 per QALY, while the upper limit is $ 150,000 per QALY [15].

Quadrant III includes interventions that are cheaper and less effective than the alternatives with which they are compared. In this quadrant, there may be interventions that are less effective than others but that provide high value to the system. This is not a contradiction. Interventions whose effectiveness is somewhat lower should be adopted if they are “almost as good and much cheaper” as others, that is, when their decremental cost-effectiveness is considered acceptable [16]. Such interventions offer the opportunity to add value to the system, since the resources saved could be used to finance high-value medical interventions. Although formally no decremental cost-effectiveness thresholds have been established, an intervention that, for example, would save $ 150,000 for each lost QALY would be efficient, since such savings could be used to finance 3 QALY of $ 50,000 in quadrant I, with a net gain of 2 QALYs (3 QALYs of $ 50,000 won in quadrant I minus 1 QALY lost in quadrant III). The definition of divestment thresholds is an open research field and it should be analyzed whether these thresholds should be symmetric or not (same value in quadrant I and III).

Payment-for-value strategies should be based on the definition of high and low value provided by the cost-effectiveness plane (Fig. 1). The application of the double threshold in these quadrants allows the cost-effectiveness plane to be divided into three zones. For example, assuming the double threshold proposed by ICER ($ 50,000 and $ 150,000 per QALY), the “high value” zone would include the part of quadrant I in which the interventions had an incremental cost effectiveness below $ 50,000 per QALY, the whole quadrant II (in which the interventions would be dominant with respect to the reference one), and the part of quadrant III in which the interventions had a decremental CE exceeding $ 150,000 (save at least $ 150,000 per lost QALY). The “low value” zone would include the part of quadrant I whose interventions exceeded the threshold value of $ 150,000 per QALY gained, all quadrant IV and the area of quadrant III in which the interventions had a decremental CE below 50,000 dollars saved by lost QALY (save less than $ 50,000 per lost QALY). Finally, there would be the two zones between the low and high thresholds of quadrants I and III, in which, apart from efficiency, other contextual factors would come into play such as the existence or not of other alternatives, the severity or the prevalence of the illness. Recently, NICE has established some of its recommendations based on the decremental CE [17].

In general, the fact that one intervention is less effective than another, or that it results in more adverse effects, or costs more than another, does not necessarily imply that it is a low-value intervention. What should define low-value care is the low efficiency of interventions, and its identification should contemplate four complementary strategies: 1) eliminating ineffective interventions that entail a cost, 2) eliminating less effective and more expensive interventions (quadrant IV), 3) eliminating interventions whose cost-effectiveness exceeds the maximum cost-effectiveness threshold in quadrant I (for example, an ICER higher than $ 150,000 per QALY gained); and 4) eliminating interventions whose decremental CE is unacceptable (for example, CE less than $ 50,000 per QALY lost in quadrant III). Finding the right balance between investing in efficient interventions and divestment in inefficient interventions is an obligation of health systems that seek to maximize the value of care (6). Only in this way can resources be freed from less valuable interventions to finance innovations that generate high value for patients.

The use of cost-effectiveness analysis to identify low value services poses some challenges. First, many countries have incorporated the economic evaluation of health technologies as a basic tool for reimbursement, pricing and purchasing decisions. By considering costs and benefits from the societal or health care sector perspectives, cost effectiveness analysis is intended to inform coverage decision at population level. However, ideally, paying for value would require identifying and selecting those interventions that add value to individual patients [18, 19].

As the concept of value is a continuum and not an absolute [20], conventional population-based cost-effectiveness analysis fails to capture the value of the interventions at the individual level. Medical services are rarely ineffective or unsafe for all patients, and under all circumstances, in that way an intervention that has demonstrated low value to the average patient may add important value to certain individual patients [21, 22]. By taking into account inter individual heterogeneity in biological, psychological and and socioeconomic factors [23], physicians could play a role in the clinical individualization of interventions, including making decisions about cost-effectiveness on a case by case basis [24].

However, perhaps it is not realistic to let clinicians analyze whether an intervention that is inefficient for the population is efficient for specific individual patients. Apart from the complexity of the process, countries that have incorporated the economic evaluation of health interventions into their reimbursement decisions usually eliminate from their formularies those interventions that are not efficient for the population. A more realistic way to personalize cost-effectiveness decisions is through the increasingly detailed subgroup analyzes included in the economic evaluations of health interventions. Furthermore, some authors have proposed to individualize cost-effectiveness analysis by providing additional metrics oriented to include the per person net benefit and cost, subgroup ICER estimates for observed measured sources of heterogeneity, and distributions of outcomes and costs for unknown or unmeasured sources of heterogeneity [25]. Although the proposal is very attractive, it is technically complex and has never been implemented. Anyway, the development of methods to capturing the value and cost of a clinical service to individual patients should be encouraged in order to move from population to individual value-based decisions.

Second, approaches to reducing low value care interventions frequently emphasize the need for a shared decision-making process between the clinician and the patient regarding the use of a specific potentially-low-value service. The development of communication education modules to aid physicians in conversations with their patients about overuse and unnecessary medical tests and procedures should be promoted [26].

Finally, many interventions have been included in the low value lists because there is no evidence about their effectiveness. It seems logical that an intervention whose effectiveness has not been evaluated is provisionally considered as “low value”, but this should not be an obstacle to carry out studies aimed at determining its effectiveness, since the absence of evidence on effectiveness is not same as the evidence of ineffectiveness [27].

## Conclusions

Incremental cost-effectiveness rather than incremental effectiveness should be the main criterion to assess the value of health care services and interventions. Although “low value care” lists are based on the incremental effectiveness of the interventions, a focus on effectiveness may generate an inefficient use of resources, as a very effective intervention is not necessarily an efficient intervention, and a low effective intervention is not always an inefficient intervention. Reducing low value care should include three complementary strategies: eliminating ineffective interventions that entail a cost; eliminating interventions whose cost is higher and whose effectiveness is lower than that of other options (quadrant IV); and eliminating interventions whose incremental or decremental cost-effectiveness is unacceptable in quadrants I and III, respectively.

At the same time, it is important to start moving from population to individual incremental cost-effectiveness analysis. The development of patient-oriented research is contributing to assess heterogeneity in the response to medical interventions. This is a crucial step to identify and select low and high value options for subgroups and individual patients.

Finally, health care systems must evolve from “cost-centric” approaches, based exclusively on savings and a definition of “low value care” based on incremental effectiveness to “value-centric” approaches, where prioritization based on efficiency seeks the appropriate balance between investing in efficient interventions and disinvesting in inefficient ones. This change will contribute to creating a new paradigm in which pay for performance approaches will be progressively replaced by pay for value models.

## Data Availability

Not applicable.
